# The Distinct Properties of the Consecutive Disordered Regions Inside or Outside Protein Domains and Their Functional Significance

**DOI:** 10.3390/ijms221910677

**Published:** 2021-10-01

**Authors:** Huqiang Wang, Haolin Zhong, Chao Gao, Jiayin Zang, Dong Yang

**Affiliations:** State Key Laboratory of Proteomics, Beijing Proteome Research Center, National Center for Protein Sciences (Beijing), Beijing Institute of Lifeomics, Beijing 102206, China; wanghuqiang814@163.com (H.W.); zhlien@mail.nankai.edu.cn (H.Z.); gaochaohappy@126.com (C.G.); zangjiayin2005@163.com (J.Z.)

**Keywords:** consecutive disordered region, protein domain, physical–chemical property, function, protein abundance, organism complexity

## Abstract

The consecutive disordered regions (CDRs) are the basis for the formation of intrinsically disordered proteins, which contribute to various biological functions and increasing organism complexity. Previous studies have revealed that CDRs may be present inside or outside protein domains, but a comprehensive analysis of the property differences between these two types of CDRs and the proteins containing them is lacking. In this study, we investigated this issue from three viewpoints. Firstly, we found that in-domain CDRs are more hydrophilic and stable but have less stickiness and fewer post-translational modification sites compared with out-domain CDRs. Secondly, at the protein level, we found that proteins with only in-domain CDRs originated late, evolved rapidly, and had weak functional constraints, compared with the other two types of CDR-containing proteins. Proteins with only in-domain CDRs tend to be expressed spatiotemporal specifically, but they tend to have higher abundance and are more stable. Thirdly, we screened the CDR-containing protein domains that have a strong correlation with organism complexity. The CDR-containing domains tend to be evolutionarily young, or they changed from a domain without CDR to a CDR-containing domain during evolution. These results provide valuable new insights about the evolution and function of CDRs and protein domains.

## 1. Introduction

**Figure 1:**
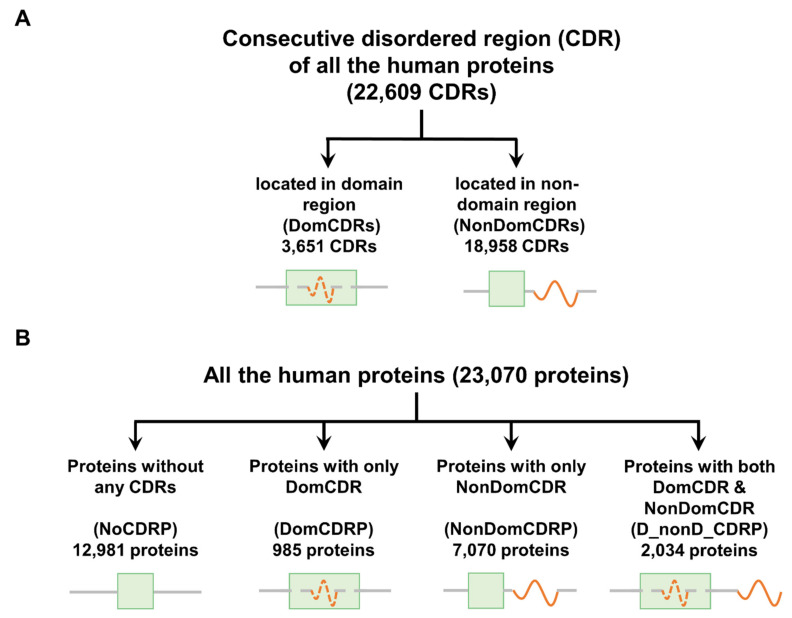
Schematic representation of the classifications of consecutive disordered region (CDR) and all the proteins. Human CDRs and proteins are used as the example in this figure. Two types of CDR were classified based on their locations relative to protein domains (**A**). Four types of proteins were classified based on the CDR types in the protein (**B**). The boxes represent the protein domains. The red dash or solid curve lines represent the CDRs inside or outside protein domains, respectively.

Intrinsically disordered proteins (IDPs) often have variable structures and functions and play an important role in the regulation of complex biological processes [1,2]. IDPs usually contain at least one consecutive disordered region (CDR) in which there are more than 30 continuous disordered residues [1,3]. The CDRs in IDPs play an important role in the transition of structure and function, contributing to the formation of functional diversity and system complexity [4]. Previous studies have found that CDRs may be located inside or outside the protein domains (referring to all Pfam-A domains in this study) [1,5,6,7]. According to the relative location, CDRs are divided into two types: CDRs inside protein domains (DomCDRs) and CDRs outside protein domains (NonDomCDRs) (Figure 1A). However, the difference between DomCDRs and NonDomCDRs is largely unknown.

At the protein level, proteins can be classified into four groups according to presence/absence and localization of CDRs within or outside the protein domains (Figure 1B): NoCDRPs (proteins without any CDRs), DomCDRPs (proteins with only DomCDRs), NonDomCDRPs (proteins with only NonDomCDRs), D_nonD_CDRPs (proteins with both DomCDRs and NonDomCDRs). Previous studies have mainly focused on the functional properties of IDPs compared with structured proteins [8,9], but the difference among different types of IDPs has rarely been studied. In this study, we will explore the differential characteristics of the four categories of proteins regarding evolution, function, expression, and stability.

CDR-containing protein domains are an important contributor to the formation of organism complexity, since there is a significant positive correlation between the proportion of CDR-containing domain families and the organism complexity as measured by cell-type number [5]. Thus, which CDR-containing protein domains are related to the formation of biological complexity? What are the characteristics of their evolutionary origins? These questions are also the focus of this study.

## 2. Results

### 2.1. Classification of CDRs and Proteins

A genome-wide analysis identifying protein domains and intrinsically disordered regions in all the proteins was performed. Taking human data as an example, we first identified 22,609 consecutive disordered regions (CDRs) with at least 30 disordered amino acid residues and classified them into two types, DomCDRs and NonDomCDRs, based on their position relative to the protein domains (Figure 1A). DomCDRs account for about 16.1% of all CDRs. Furthermore, all the proteins were divided into four categories according to the CDR they contain (Figure 1B). About half of the proteins have CDRs. Among them, more than two-thirds are NonDomCDRPs, while only a small fraction (less than10%) are DomCDRPs. Protein domains containing CDRs may play an important role in increasing organism complexity [5], indicating that DomCDRs and DomCDRPs may have special functions. Thus, the property differences among these two types of CDRs and four types of proteins were investigated in this study.

### 2.2. The Distinct Physicochemical and Interaction Properties of Two Types of CDRs

**Figure 2:**
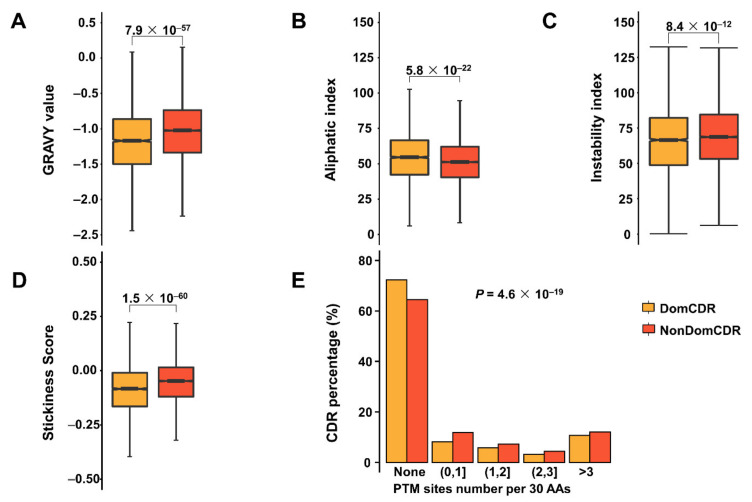
The difference between two types of CDR in physical–chemical properties (**A**–**D**) and post-translational modification site numbers (**E**). GRAVY (Grand Average of Hydropathy) value indicates the average hydropathy value of a peptide or protein. Boxplots show the GRAVY value (**A**), aliphatic index (**B**), instability index (**C**) and stickiness score (**D**) of the two types of CDR. In the boxplots, the values of the upper and lower quartiles are indicated as the upper and lower edges of the box, and the median values are indicated as a bar in the box. The differences were examined by the Wilcoxon rank sum test. The corrected *p* values are shown at the top of each panel. The PTM site numbers were compared between the two types of CDRs (**E**). The PTM site numbers per 30 amino acids (AAs) were classified into four groups and the percentage of CDRs in each group was calculated. The difference was tested with the chi-square test.

We compared the physicochemical properties of DomCDRs and NonDomCDRs and found significant differences in their hydrophobicity, aliphatic property, and instability (Figure 2A–C, Table S1). Hydrophobicity is one of the most basic physicochemical properties of peptides or proteins. We used the GRAVY (Grand Average of Hydropathy) values [10] to represent the average hydropathy value of a peptide or protein. From the result of the hydrophobicity analysis, we found that both DomCDRs and NonDomCDRs have negative GRAVY values, which means that both of them are hydrophilic. However, DomCDRs have lower hydrophobicity scores, indicating they are more likely exposed on the protein surface (Figure 2A).

The aliphatic index of a protein/peptide is defined as the relative volume occupied by aliphatic side chains (alanine, valine, isoleucine, and leucine) [11]. It may be regarded as a positive factor for the increase in the thermostability of globular proteins. The higher aliphatic index of DomCDRs (Figure 2B) indicated that they contain more aliphatic side chains and are more stable [11]. The instability index provides an estimate of the stability of one protein/peptide based on its amino acid composition [12,13]. A protein/peptide whose instability index is smaller than 40 is predicted as stable. Most of the two types of CDR have an instability index above 40, indicating both of them may be unstable. DomCDRs have a relatively smaller instability index (Figure 2C), meaning they are more stable than NonDomCDRs.

To further uncover the functional difference between the two types of CDR, we compared the stickiness (interaction propensity) scores [14,15] and the PTM (post-translational modification) site numbers between them. Here, stickiness was calculated as the average stickiness score based on the scoring matrix [15]. Lower stickiness scores mean the DomCDRs avoid promiscuous interactions (Figure 2D, Table S1), while at the same time, DomCDRs showed fewer PTM sites compared with NonDomCDRs (Figure 2E, Table S1). These results suggested that DomCDRs have fewer interactions with other proteins, including the enzymes that catalyze PTM. These characteristics may protect the DomCDRs from abnormal aggregation and degradation, which was consistent with their higher stability as compared with NonDomCDRs.

### 2.3. The Distinct Evolutionary and Functional Characteristics of the Four Protein Categories Classified Based on the Two Types of CDR

To compare the characteristics of the four protein categories, we first calculated the protein structural disorder ratio (PSDR), the percentage of the disordered amino acid residues in a protein (Figure S1) [5,16,17]. As expected, NoCDRPs have the lowest PSDR values protein and D_nonD_CDRPs, which have two types of CDR, have the highest overall disorder ratio. It is interesting that the overall disorder ratio of DomCDRPs is higher than that of NonDomCDRPs. The reason for this phenomenon may be that the DomCDRPs contain many disordered amino acids but no long continuous disordered regions outside protein domain, while NonDomCDRPs only contain long consecutive disordered regions outside the protein domains. Therefore, the PSDR values of the NonDomCDRPs are not as high as those of DomCDRPs.

**Figure 3:**
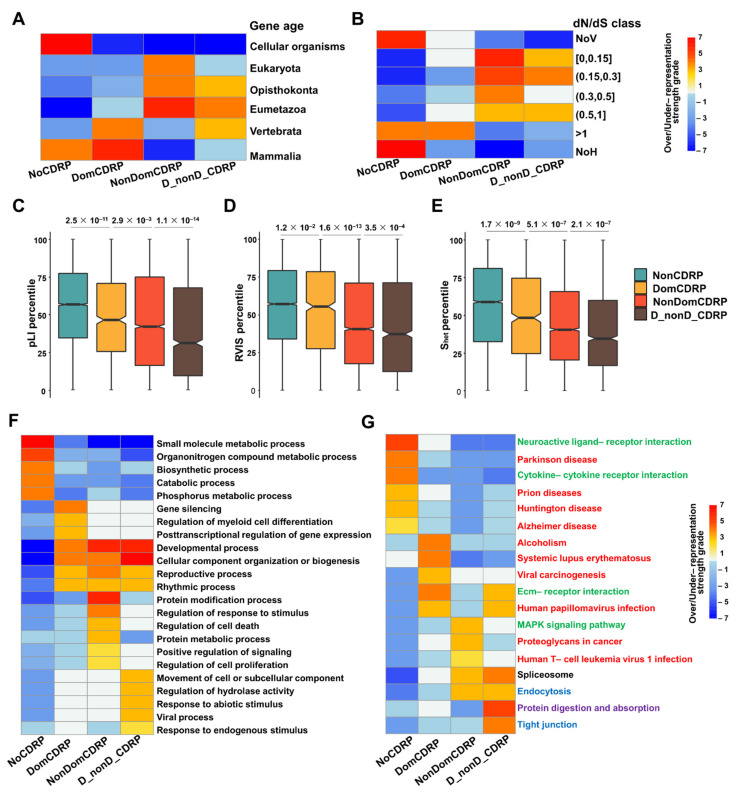
The evolutionary and functional characteristics of the four classes of proteins. Over- or under-representation strengths of the four protein categories in gene-age (**A**) or dN/dS (**B**) classes. NoV means no variation between the orthologs of human and chimpanzee. NoH means no homologs between human and chimpanzee. (**C**–**E**) Comparison of the tolerance to functional variation among the four types of proteins. The residual variation intolerance score (RVIS), the probability of being intolerant to loss-of-function mutations (pLI), and the selection against heterozygous loss of gene function (S_het_) scores are further transformed into percentiles. Significance was measured by the Wilcoxon rank sum test and *p*-values were adjusted by step-down Bonferroni. (**F**,**G**) Over- or under-representation analysis of biological process (BP) and KEGG pathways for the four types of proteins. The over- or under-representation strengths (**A**,**B**,**F**,**G**) are represented by −log (p) or log (p), respectively. Heat maps show the grades of over- or under-representation strengths, scoped from −7 to 7. Red terms belong to the “Human Diseases” class; green terms belong to “Environmental Information Processing” class; blue terms belong to the “Cellular Processes” class; purple terms belong to the “Organismal Systems” class and the black term belongs to the “Genetic Information Processing” class.

The evolutionary origin time and evolutionary rate of genes are important indicators of their functional specificity and selection pressure in the process of evolution, respectively. Gene origin time, also known as gene age, represents the phylogeny scope (the range of species) in which one gene has its homologs. Over- or under-representation analysis of the four protein categories in each gene age class was performed Interestingly, NoCDRPs are either very young or very old. DomCDRPs are only enriched in the two young gene-age classes (Vertebrata and Mammalia), while other types of CDRPs were not enriched in the youngest gene-age classes (Mammalia) (Figure 3A, Table S2). We further tested the evolution rates of the four categories of proteins. Some NoCDRPs and DomCDRPs turn out to evolve faster than other proteins, indicating they are under positive selection (Figure 3B). To further investigate their differences in functional constrains, three different measurements of functional constraint were used in our study: the residual variation intolerance score (RVIS) [18], the probability of being intolerant to loss-of-function mutations (pLI) [18] and the selection against heterozygous loss of gene function (S_het_) [19]. The greater the values of these three parameters, the stronger the tolerance to gene variation, i.e., the lower functional constraints. As a result, CDRPs—especially D_nonD_CDRPs—were under strong functional constraint, while DomCDRPs showed the lowest functional constraint among CDRPs (Figure 3C–E).

These results revealed that DomCDRPs originated later and evolved faster, compared with other CDRPs. They should have distinct functions. Thus, we then conducted over- or under-representation analysis of biological process terms in the Gene Ontology (GO) and KEGG pathways to discover the functional characteristics of the four protein categories. GO analysis showed that NoCDRPs tend to be involved in such molecular metabolisms as biosynthetic or catabolic processes. On the other hand, all CDRPs are highly involved in important regulation of life processes, such as developmental, reproductive, and rhythmic processes. Specifically, DomCDRPs are enriched in genetic information processes such as gene silencing, post-transcriptional regulation of gene expression and immune-related processes such as the regulation of myeloid cell differentiation. NonDomCDRPs are more involved in the regulation of cellular processes, cell proliferation, cell death, signaling, or protein modification. D_nonD_CDRPs mainly focus on the specific kinds of responses to stimulus, such as responses to endogenous stimulus, response to abiotic stimulus, regulation of hydrolase activity and viral processes (Figure 3F, Table S3A).

In the KEGG pathway analysis, NoCDRPs are found to be more involved in neurodegenerative diseases such as Parkinson’s disease, Huntington’s disease, Alzheimer’s disease and neuron-related signaling molecules and interaction pathway–neuroactive ligand-receptor interaction, while CDRPs are more involved in infectious disease and cancer. Specifically, DomCDRPs are found to be highly enriched in systemic lupus erythematosus and alcoholism. Similar to D_nonD_CDRPs, DomCDRPs are also enriched in ECM–receptor interaction and human papillomavirus infection (Figure 3G, Table S3B).

### 2.4. DomCDRPs Are Not Over-Represented in Certain States and Tend to Be Expressed with Spatiotemporal Specificity Compared with NonDomCDRPs

**Figure 4:**
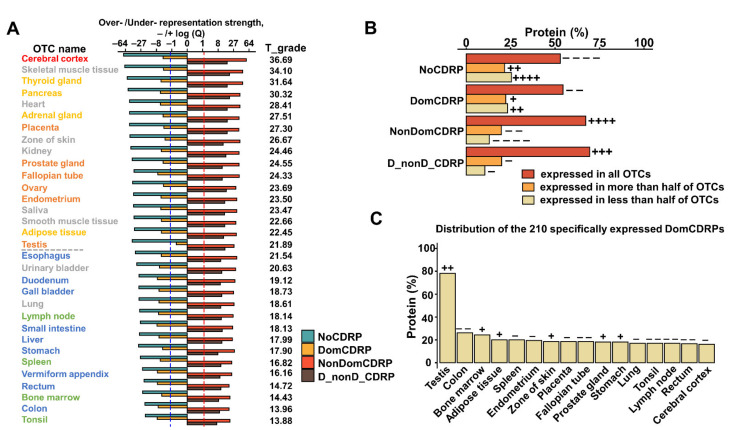
The expression patterns of four types of proteins in 32 organ, tissue or cell types (OTCs). (**A**) Over- or under-representation strengths of each protein category in 32 OTCs. Over- and under- representation are represented by -log(Q) or log(Q), respectively (see Methods for details). The red/blue dashed line represents the –/+log(Q) value corresponding to significant over- or under-representation. T_grade represents the strength for its consistence with the general over- or underrepresentation trends of most OTCs. Red OTC name belongs to the “nervous system”; gold OTC names belong to the “endocrine System”; orange OTC names belong to the “reproductive system”; blue OTC names belong to the “digestive system”; green OTC names belong to the “immune system” and grey OTC names belong to other systems. The grey dashed line separates two groups of OTCs with stronger/weaker over-/under-representation strengths. (**B**) Percentage of proteins in three groups with different OTC-specific grades. (**C**) Distribution of the 210 specifically expressed DomCDRPs. Selected OTCs are the OTCs with top 16 DomCDRPs’ expression percentages. Fisher’s exact test was used to calculate the over- or under-representation strength, and the *P* values were corrected using the Benjamini–Hochberg method. The results are Q values. ++/− − represents *Q* ≤ 0.05, +++ represents *Q* ≤1 × 10^–10^; ++++/− − − − represents *Q* ≤ 1 × 10^–50^.

The gene expression patten provides important clues for the understanding of the protein functions. To further explore the functional difference among the four categories of proteins, we conducted an over-representation analysis to investigate their expression pattern. It is obvious that NonDomCDRPs and D_nonD_CDRPs are significantly over-represented in each OTC (organ, tissues, or cell type) and developmental stage of seven human organs, whereas DomCDRPs and NoCDRPs are usually under-represented in each state (Figure 4A, Table S4A, Figure S2A). This result indicates that a large fraction (much more than the proportional theoretical number) of CDRPs (proteins containing CDRs) are usually expressed at given physiological states; however, the expression number of DomCDRPs is much less than the expected number. That is, only a small number of DomCDRPs are utilized in certain states. Interestingly, OTCs belonging to nervous, endocrine and reproductive systems have a stronger over/under-representation strength, while the trends in the OTCs belonging to digestive and immune systems are relatively weaker (Figure 4A), indicating there are regular differences in the utilization of the four protein cateorgaries in different types of OTCs.

The spatiotemporal specificity analysis confirmed the above results from another viewpoint. All the proteins were divided into three groups with different expression widths (Figure 4B, Table S4B, Figure S2B), and the percentage of proteins in these three groups within each of the four protein categories was calculated. Consistently, DomCDRPs tend to be expressed in specific OTCs and stages compared with NonDomCDRPs. In detail. the specifically expressed DomCDRPs are significantly enriched in adult testis (Figure 4C, Table S4C), fetal testis 12–13 weeks post conception (Table S4D), fetal heart 8 weeks post conception and fetal ovary 18 weeks post conception (Table S4D). Altogether, these results indicate that DomCDRPs tend to function in specific spatiotemporal states.

### 2.5. DomCDRPs Have High Abundance and Long Half-Life Time

**Figure 5:**
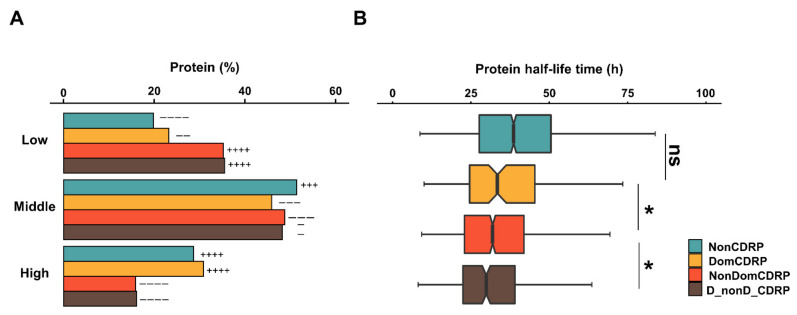
The abundance (**A**) and the half-life time (**B**) of four types of proteins. Proteins were classified into three groups (low, middle, and high, see Figure S3 for detailed information). (**A**) Histograms show the percentages of the three groups of proteins in each of the four types. Fisher’s exact test was used to calculate the over- or under-representation strength. − − represents *q* ≤ 0.05, +++/− − − represents *q* ≤ 1 × 10^−10^; ++++/− − − − represents *q* ≤ 1 × 10^–50^. (**B**) Box plots show the half-life time values of the four types of proteins. The values of the upper and lower quartiles are indicated as upper and lower edges of the box, and the median values are indicated as bars in the box. The differences are measured by Wilcoxon rank sum test and *p*-values were adjusted by step-down Bonferroni; *, *q* < 0.05.

The results related to the expression patterns mentioned above are mainly at the qualitative level (focusing only on expression or not). What are the expressional characteristics of these four types of proteins at the quantitative level? To answer this question, we performed analyses focusing on protein abundances and half-life times. Protein abundances were taken from the quantitative proteome data of 58 human OTCs [20,21]. Proteins were classified in three grades (high, middle and low) according to their abundance (Figure S3). DomCDRPs have the highest proportion of highly abundant proteins, whereas the other two types of CDRPs tend to express at a low level (Figure 5A, Table S5A). The protein half-life time analysis is conducted to gain insights into the four categories of protein stability. We found that DomCDRPs have a significantly longer half-life time than the other two types of CDRPs, indicating that DomCDRPs are more stable (Figure 5B, Table S5B). This result is consistent with the stability analysis of DomCDR (Figure 2B–C).

### 2.6. The Significance of DomCDRPs in Increasing Organism Complexity

**Figure 6:**
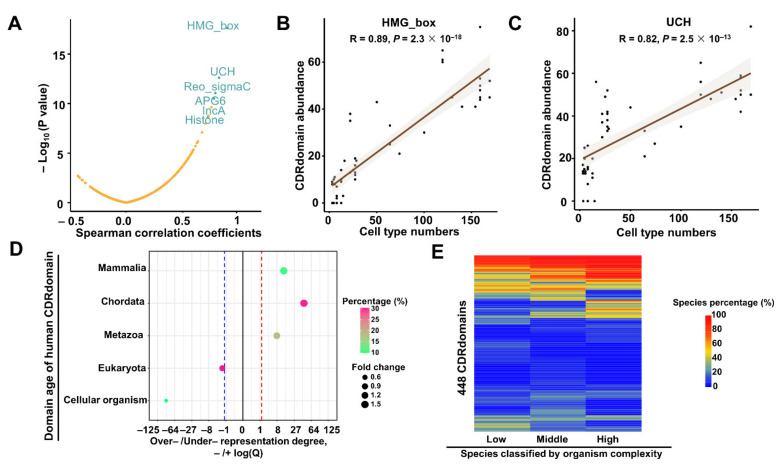
The contribution of the protein domains containing CDRs to organism complexity. (**A**) The domain abundance is the number of the genes encoding proteins containing this domain in one genome. The Spearman correlation coefficients (R values) were calculated between each CDR domain abundance and the organism complexity measured by cell-type number. The scatter plot shows the correlation between the Spearman correlation coefficients and *p*-values. The names of six representative protein domains (R > 0.7, the number of species where the domain has no CDR is less than 10) are shown. Two examples HMG_box (**B**) and UCH (**C**), are shown by the scatters representing the correlation between domain abundance in one genome and cell-type number of species. (**D**) Over- or under-representation strengths of the human CDRdomains in five domain age grades. Over- and under- representation are represented by –log(P) or log(P), respectively (see Methods for details). The red/blue dashed line represents the –/+log(P) value corresponding to significant over- or under-representation. The percentage of the domains with a certain age is marked as gradual colors. Fold change, represented by the size of the circle, means the ratio of the number of domains of each age grade to the proportional expected value. (**E**) Clustered heat map shows the percentages of the species where the domain contains a CDR in each complexity group for the 448 CDRdomains.

Our previous study revealed that protein domains containing CDRs (herein called as “CDRdomains”) may play important roles in increasing organism complexity during evolution. Thus, in this study, we aimed to discover those CDRdomains with a significant correlation with organism complexity measured by the cell-type number of the species. We identified 448 protein domains shared in 51 species (Table S6A) and counted the number of proteins containing these domains in each genome, which was called domain abundance. Then, the correlation coefficients between domain abundance and organism complexity were calculated. As a result, we found 16 CDRdomains with strong correlation with organism complexity (|R| > 0.6, *p* < 0.05), such as HMG box and UCH (Figure 6A–C, Table S6B). Then, we analyzed the domain age of the CDRdomains in human proteins. CDRdomains in human proteins were found to be very young (Figure 6D). Additionally, lots of domains tend to have no CDRs in low organism complexity grade, while have CDRs in high organism complexity grade (Figure 6E, Table S6C). For example, the SNF2_N domain in yeast protein P31380 has no CDR, but in human protein HLTF displays a long CDR in the *N* terminal (Figure S4).

## 3. Discussion

In this study, we focused on the differences between CDRs inside and outside protein domains and their functional relevance to the proteins containing these two types of CDRs. Firstly, we found that DomCDRs are more hydrophilic and stable, but have less stickiness and fewer PTM sites compared with NonDomCDRs. Secondly, at the protein level, DomCDRPs originated late, evolved rapidly, and have weak functional constraints, compared with NonDomCDRPs and D_nonD_CDRPs. DomCDRPs tend to be expressed with spatiotemporal specificity, but they have higher abundance and are more stable. Thirdly, we screened the CDR-containing protein domains that have a strong correlation with organism complexity. There are two reasons for the increasing proportion of CDR-containing domains during evolution. One is that the CDR-containing domains tend to be evolutionarily young, that is, many newly emerging domains have CDRs. Second, in simple organisms, some protein domains have no CDRs, while in complex organisms, the domains belonging to the same family have CDRs, which indicates that the disorder degree of this type of domain has changed during evolution. Here, we further explored the possible causes and mechanisms of some interesting phenomena found in this study.

DomCDRs have stronger hydrophilicity, indicating they tend to be exposed on the protein surface, but there is a relatively small possibility of interaction with other molecules. Here, we speculate on the possible reasons for this paradox. Firstly, DomCDRs have a higher hydrophilicity, which may be due to the functional requirement of the CDRs in domains. They need higher hydrophilicity to be outside the global domain. However, CDRs outside domains do not t need to be hydrophilic because they can easily interact with other molecules. Secondly, as CDRs are flexible peptide chains that are often involved in non-specific interactions, such loose regulation may lead to serious consequences. The promiscuous interactions triggered by CDRs might be toxic or competitively inhibit normal interactions, resulting in disturbance, or even destruction of cellular homeostasis. Therefore, we speculate that there are certain sequence characteristics of DomCDRs prevent them from non-functional contact with other proteins. An effective index evaluating peptide interaction propensity is amino acid stickiness, as reported by [14,15]. Amino acids with higher stickiness scores appear more frequently on the surface of proteins; thus, we can measure the interaction propensity of a given protein segment by calculating its average stickiness score. We calculated the average stickiness of DomCDRs and NonDomCDRs, and DomCDRs had significantly lower average stickiness than NonDomCDRs (Figure 2D). The stringently controlled abundance of D_nonD_CDRPs can explain this result because the higher stickiness of DomCDRs or NonDomCDRs in D_nonD_CDRPs may not be able to cause irreversible disturbance to cellular processes if the protein they locate in is rapidly degraded.

Our work highlighted that DomCDRPs are the youngest CDRPs, evolve fastest, function in specific spatiotemporal states (such as testis), and have a high level of abundance and lower function constraint. CDRdomains are also very young. All together, these results indicated that DomCDRPs may be a special subset of proteins that need a more detailed investigation of their specific functions. There must be inherent relationship between the special properties of DomCDR and the specific expression and functional characteristics of DomCDRPs, which need further and deep studies.

As for the mechanism of the increasing organism complexity, one of our previous studies proposed a “two-level” model that complex and young genes contribute to organismal complexity at two different levels (complex genes contribute to the complexity of individual proteomes in certain states, whereas young genes contribute to the diversity of proteomes in different spatiotemporal states) [22,23]. Moreover, we also found the proportion of CDRdomain families have a significant positive correlation with organism complexity, indicating CDRdomain is an important contributing factor of organism complexity [5]. In this study, we further found that DomCDRPs tend to be young and expressed specifically, suggesting CDRdomains (in DomCDRPs) mainly contribute to the diversity of proteomes in different spatiotemporal states. That is, we found the main way in which CDRdomain promotes organism complexity.

Notably, 3D nature of the polypeptide chain was not taken into account in the design of this study because of the low 3D structure annotation percentage. For example, only 35.6% human proteins in SwissProt database have 3D structure annotation. The comparison of the 3D nature of the CDRs inside/outside protein domains will be done in the future.

This study provides a systematic analysis of two types of CDR, four categories of proteins and CDRdomains that may be useful to further studies on CDR and domain evolution and function analysis. In future, we will focus on theses CDRdomains’ roles in diseases such as cancer to gain a more specific biology related understanding of CDRs and domains.

## 4. Materials and Methods

### 4.1. Datasets

The protein sequences were retrieved from the Ensembl database [24] (V90, ftp://ftp.ensembl.org/pub/release-90/fasta/, Access date: 23 April 2021. PTM data were download from iPTMnet [25] (https://research.bioinformatics.udel.edu/iptmnet/download, Access date: 28 July 2021). The RNA-seq data used in Figure 4 and Figure S2 were from the ArrayExpress database (E-MTAB-2836 [26] and E-MTAB-6814 [27], respectively). 

The published human quantitative proteome data [20,21] of 58 histologically normal human organ, tissue or cell types (OTCs) or body fluid were used in the analysis of protein abundance. The half-life time data were downloaded from the supplement file of this research [28].

### 4.2. Identification of Protein Domains, CDR and Protein Classification

Domains were identified with the HMMER-scan program [29] to search the Pfam databases [6]. All Pfam-A domain entries were used to identify protein domains in each protein. The full sequence E value and C-E value were kept at less than 0.01. The overlapped hits with smaller E value were kept. 

CDR is defined as a long-disordered region with over 30 disordered residues [30,31,32]. The disordered amino acid prediction was performed in Iupred2A [33]. According to the relative location of the CDR and protein domain, the CDRs were classified into two types, DomCDRs or NonDomCDRs, representing the CDRs locating inside or outside a protein domain, respectively (Figure 1A). If a CDR overlapped with a domain and the length of overlapping was over 20 residues [5,34], it was considered as a DomCDR, and if the length of the CDR in a nondomain region was over 20 residues, it was otherwise considered as a NonDomCDR. 

All the proteins were classified into four categories based on the CDRs they had (Figure 1B): NoCDRPs (proteins without any CDRs), DomCDRPs (proteins with only DomCDRs), NonDomCDRPs (proteins with only NonDomCDRs), D_nonD_CDRPs (proteins with both DomCDRs and NonDomCDRs).

### 4.3. The Physico-Chemical and Interaction Properties Analysis of CDR

GRAVY score, aliphatic index and instability index were referenced from the Protein Identification and Analysis Tools chapter on the ExPASy Server [35]. The stickiness scores were referenced from two previous research studies [14,15]. 

GRAVY (Grand Average of Hydropathy) values [10] is defined as the average hydropathy value of a peptide or protein. Proteins with negative GRAVY values are hydrophilic and positive values mean they are hydrophobic.

The aliphatic index of a protein is defined as the relative volume occupied by aliphatic side chains (alanine, valine, isoleucine, and leucine). It may be regarded as a positive factor for the increase in the thermostability of globular proteins [11].

The instability index provided an estimate of the stability of one protein or peptide [12,13]. This index predicts the stability of a protein/peptide based on its amino acid composition. A protein/peptide whose instability index is smaller than 40 is predicted as stable, a value above 40 predicts that the protein/peptide may be unstable.

Stickiness is an alternative term for ‘interface propensity’ of amino acids, which was obtained from a previous study [14]. Amino-acid interface propensities were calculated by the log ratio of the frequency at the interface-core versus at the surface, based on a non-redundant set of proteins of known structure [15]. The greater the frequency of an amino acid appearing on the contact surface between different proteins, the higher the stickiness score of the amino acid. 

All the physicochemical and interaction properties value calculations were performed in Python script.

### 4.4. Evolutionary Features

Gene age: the human protein gene age information was from a consensus gene-age dataset that integrated 13 orthology inference algorithm [36].

dN/dS: The number of synonymous substitutions per synonymous site (dS) and the number of nonsynonymous substitutions per nonsynonymous site (dN) between the ortholog pairs of *H. sapiens* (human) and *P. troglodytes* (chimpanzee) were retrieved using BioMart [37] from the Ensembl database (http://aug2017.archive.ensembl.org/biomart/martview/, Access date: 28 February 2020). The ratio of dN and dS (dN/dS) values was used to represent the evolutionary rate of one protein.

Domain age: as in our previous studies [5,22,23], the domain age was assigned according to its phylogenetic distribution using the taxonomy information in the Pfam database (http://www.sanger.ac.uk/Software/Pfam/ftp.shtml, Access date: 28 February 2019). 

Organism complexity was measured by the cell-type number in a species [5,17,22,38].

### 4.5. The Functional Constraint Measurements

Three different measurements of functional constraint were used in our study. (1) the residual variation intolerance score (RVIS); (2) the probability of being intolerant to loss-of-function mutations (pLI); and (3) the selection against heterozygous loss of gene function (S_het_). We obtained the pLI and RVIS percentile values and Shet scores from the published data (pLI and RVIS: [18]; Shet: [19]). For these three parameters, the greater the value, the stronger the tolerance to gene variation, that is, the weaker the functional constraint, and vice versa.

### 4.6. Proteome Data Reprocessing and Normalization

The raw data files were researched using MaxQuant (Version1.5.3.17) [39] against the UniProt database (Homo sapiens FASTA database updated on 4 April 2015) to obtain the protein identification and the intensity-based absolute quantification (iBAQ) values. Data were searched with a 20 p.p.m. precursor ion tolerance window for the first search, a 4.5 p.p.m. precursor ion tolerance window for the main search, and a 20 p.p.m. fragment ion tolerance window. For each peptide, up to two missed cleavages were allowed. Cysteine carboxyamidomethylation was specified as a static modification, and the oxidation of methionine residue and the acetyl (protein-N) were allowed as variable modifications for each sample set. Reverse decoy databases were included for all of the searches to estimate the false discovery rate (FDR), and the data were filtered using an FDR of 1%. Each protein’s iBAQ value was divided by the sum of the iBAQ values of all the identified proteins in each sample, and the result value was multiplied by 106. Reverse and contaminant proteins were ruled out in the calculation of the sum of the iBAQ values of all the identified proteins.

### 4.7. The Correlation between CDRdomain and Organism Complexity

We used the number of proteins with at least one predicted hit in the respective Pfam domain to measure the CDRdomain abundance [38]. We identified domains shared by 51 species and identified those with a CDR in at least one species. Then, we calculated CDRdomain abundance and its correlation with the organism complexity, measured by cell-type number. 

A total of 51 species then were then categorized into three groups according to the cell-type numbers. The ‘Low’ group contained 15 species with the fewest cell-type numbers, and the ‘High’ group contained 15 species with the most cell-type numbers and the ‘Middle’ group contained the remaining 21 species. For each group, the percentage of species that contained this CDRdomain was calculated.

### 4.8. General Approach for Statistical Analysis

Over- or under-representation analyses in Figure 3, Figure 4, Figure 5 and Figure 6 was based on the hypergeometric distribution model, and the Benjamini–Hochberg method was used to correct the *p*-value [40]. The logarithm of the *p*-value (log P), positive or negative, was used to represent the strength of the over- or under-representation of proteins containing certain domain characters. When the heat maps were used to represent the over- or under-representation strengths, the values of ±log(P) were transformed into 14 grades (−7 to +7) similar to our previous analysis [5]: log(P) ≤ −30; −6, −30 < log(P) ≤ −15; −5, −15 < log(P) ≤ −10; −4, −10 < log(P) ≤ −5; −3, −5 < log(P) ≤ −2; −2, −2 < log(P) ≤ −1.301; −0.25, −1.301 < log(P) ≤ 0; 0.25, 0 < −log(P) < 1.301; 2, 1.301 ≤ −log(P) < 2; 3, 2 ≤ −log(P) < 5; 4, 5 ≤ −log(P) < 10; 5, 10 ≤ −log(P) < 15; 6, 15≤ −log(P) < 30; 7, −log(P) ≥ 30.

T_grade represented in Figure 4A was defined as the average log P among the different groups of proteins (we define log P as positive value if the trend is consistent with the general trend, otherwise, we define it as negative value) [23].

Wilcoxon rank sum test and chi-square test were performed by the R function compare_means and chisq.test, respectively. Spearman correlation analysis was performed by the python function scipy.stats.spearmanr.

## Data Availability

All data generated or analyzed in this study are included in this published article and the Supplementary Information Files. Supplementary Figures and Tables are available at IJMS online.

## Supplementary Materials

The following are available online at www.mdpi.com/1422-0067/22/19/10677/s1.
