# Supplementary material for: The Distinct Properties of the Consecutive Disordered Regions Inside or Outside Protein Domains and Their Functional Significance
Source: Int J Mol Sci. 2021 Oct 1;22(19):10677. doi: 10.3390/ijms221910677 (PMC8508753; doi:10.3390/ijms221910677)
Supplement: Supplementary file 1 [file ijms-22-10677-s001.zip › ijms-1385902-supplementary proof.pdf]

International Journal of
Molecular Sciences

MDPI

Article

The Distinct Properties of the Consecutive Disordered Regions Inside or Outside Protein Domains and Their Functional Significance

Huqiang Wang, Haolin Zhong, Chao Gao, Jiayin Zang and Dong Yang *

State Key Laboratory of Proteomics, Beijing Proteome Research Center, National Center for Protein Sciences (Beijing), Beijing Institute of Lifeomics, Beijing 102206, China; wanghuqiang814@163.com (H.W.); zhlien@mail.nankai.edu.cn (H.Z.); gaochaohappy@126.com(C.G.); zangjiayin2005@163.com (J.Z.)

* Correspondence: yangdong@ncpsb.org.cn; Tel.: 8610-61777051.

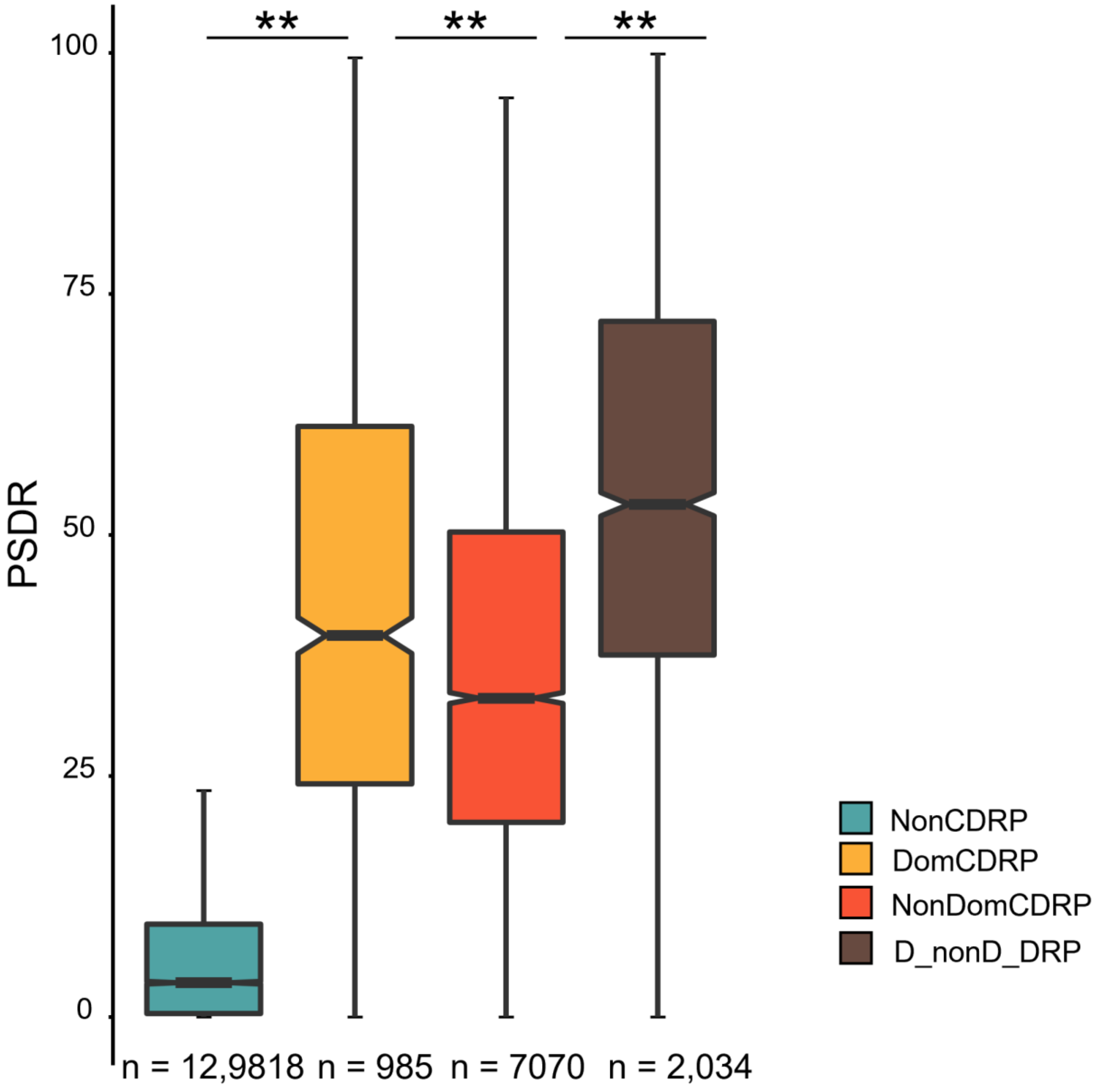

Figure S1. PSDR ratios of four different CDRPs. Four categories of proteins have significant difference in PSDR (protein structural disorder ratio). In boxplots, the values of upper and lower quartile are indicated as upper and lower edges of

the box, and the median values are indicated as a bar in the box. **, $p \le 0.01$. The differences are examined by Wilcoxon rank sum test.

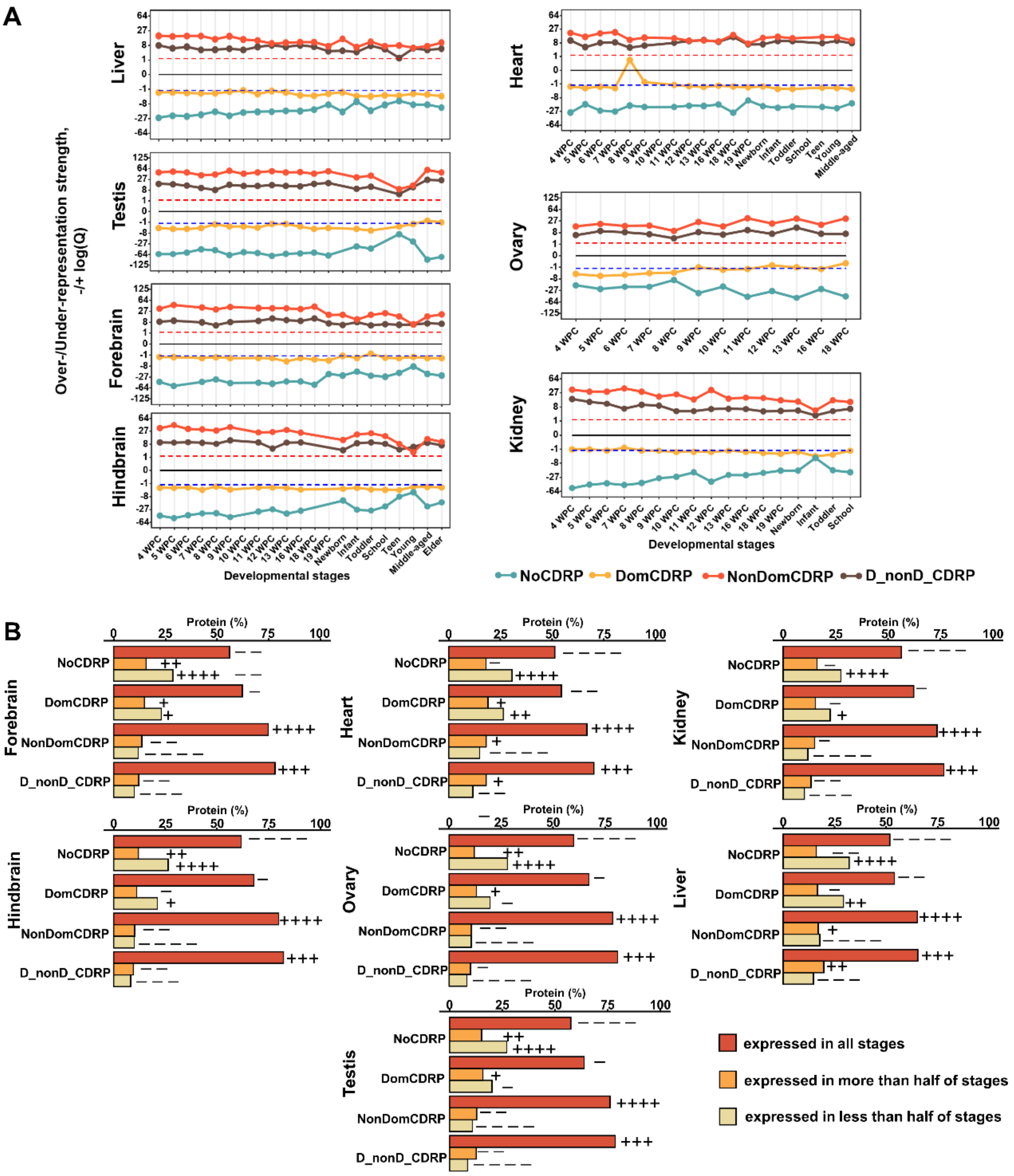

Figure S2. The expression pattern of four different CDRPs in the development of seven organs (related to figure 4). (**A**) Over- or under-representation strengths of each protein category during development of human seven organs. Over- and under- representation are represented by -log(Q) or log(Q), respectively (see Methods for details), and the *P* values were corrected using the Benjamini–Hochberg method. The results are Q values. The red/blue dashed line represents the –/+log(Q) value corresponding to significant over- or under-representation. (**B**) Percentage of proteins in three groups with different stage-specific grades. DomCDRP also shows the tendence to express in less than half of stages. Fisher exact test was used to calculate the over- or under-representation strength. ++/– – represents $q \le 0.05$, +++/– – – represents $q \le 1 \times 10^{-10}$; ++++/– – – – represents $q \le 1 \times 10^{-50}$.

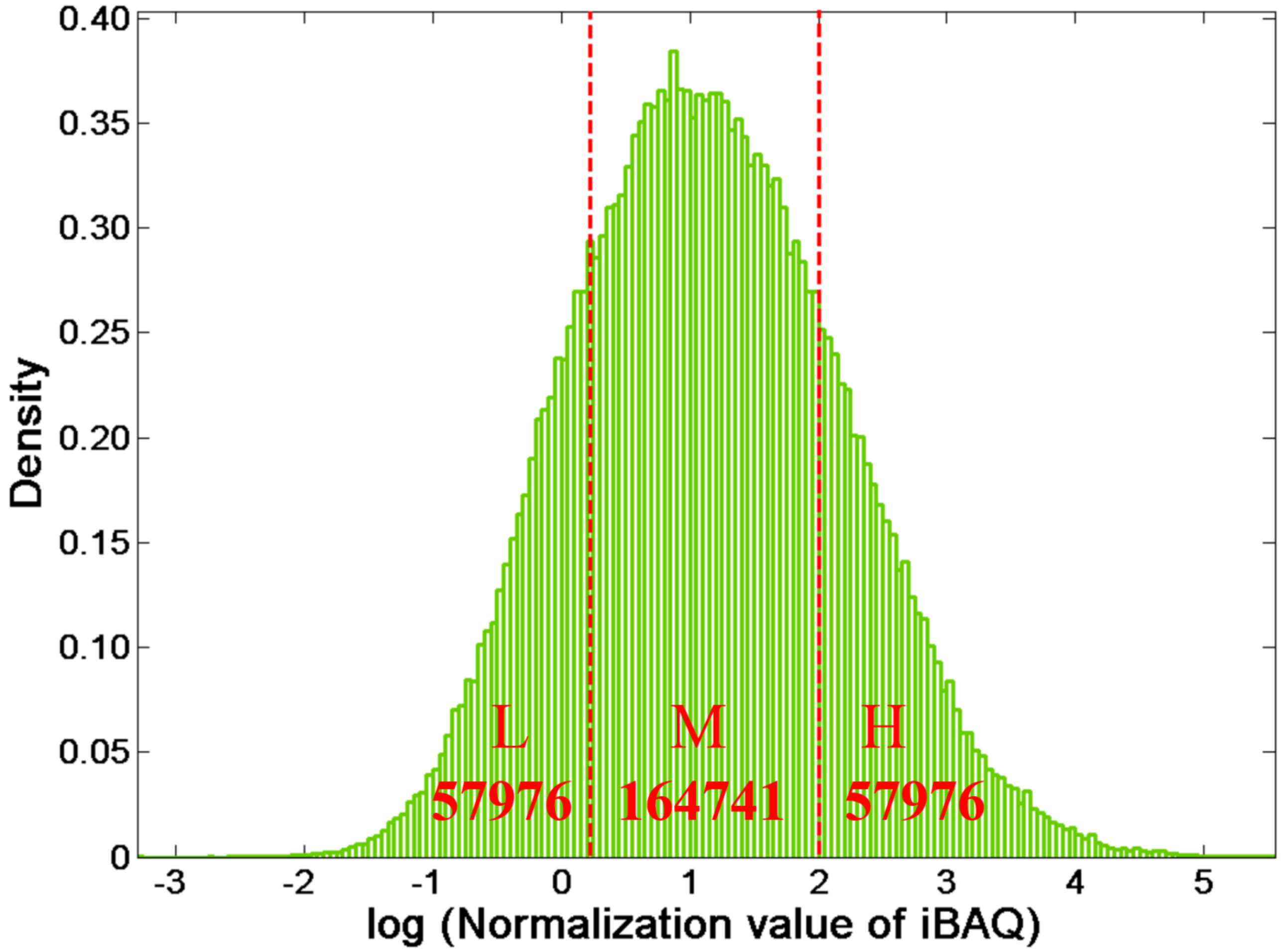

Figure S3. Protein abundance grades (related to figure 5A). The normalized iBAQ values of proteins identified in 58 OTCs are shown in the frequency diagram. Protein abundance were divided into three grades: 'high-abundance' (H), 'medium-abundance' (M) and 'low-abundance' (L). The protein numbers of each grade were shown.

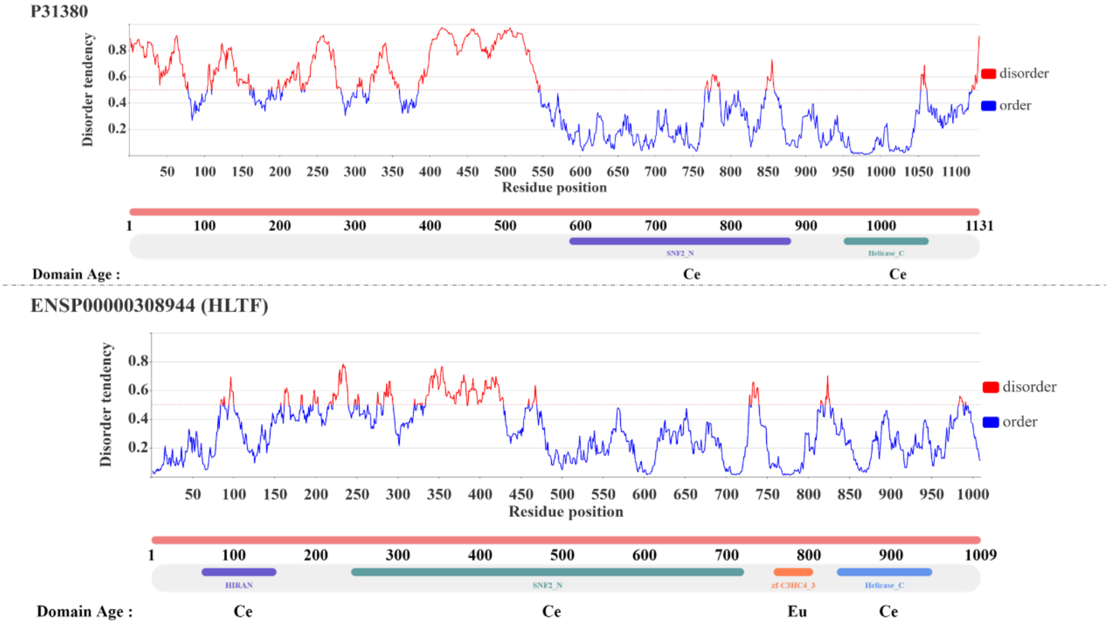

Figure S4. The SNF2_N domain in P31380 (*Saccharomyces cerevisiae)* shows no CDR and in HLTF (*Homo sapiens.*) shows CDR (related to figure 6). Some CDRdomains have no CDR region in the domain region, but in more complex species, the CDR is found in domain regions. Here is the example of SNF2_N. 46.7% low complexity species has CDR, while 81% in middle complexity species and 100% in high complexity species. The red dot (score > 0.5) indicated the residue is disordered and the bule dot indicated the residue is ordered. Ce, Cellular organism; Eu, Eukaryota.
